# Desferrioxamine Reduces Oxidative Stress in the Lung Contusion

**DOI:** 10.1155/2013/376959

**Published:** 2013-08-01

**Authors:** Umit Nusret Basaran, Suleyman Ayvaz, Burhan Aksu, Turan Karaca, Mustafa Cemek, Ihsan Karaboga, Mustafa Inan, Feyza Aksu, Mehmet Pul

**Affiliations:** ^1^Department of Pediatric Surgery, Medical Faculty, Trakya University, 22030 Edirne, Turkey; ^2^Department of Histology and Embryology, Medical Faculty, Trakya University, 22030 Edirne, Turkey; ^3^Biochemistry Division, Department of Bioengineering, Faculty of Chemical and Metallurgical Engineering, Yildiz Technical University, 34210 Istanbul, Turkey; ^4^Department of Cardiology, Edirne State Hospital, 22100 Edirne, Turkey

## Abstract

Our hypothesis in this study is that desferrioxamine (DFX) has therapeutic effects on experimental lung contusions in rats. The rats were divided into four groups (*n* = 8): control, control+DFX, contusion, and contusion+DFX. In the control+DFX and contusion+DFX groups, 100 mg/kg DFX was given intraperitoneally once a day just after the contusion and the day after the contusion. Contusions led to a meaningful rise in the malondialdehyde (MDA) level in lung tissue. MDA levels in the contusion+DFX group experienced a significant decline. Glutathione levels were significantly lower in the contusion group than in the control group and significantly higher in the contusion+DFX group. Glutathione peroxidase (GPx) and superoxide dismutase (SOD) levels in the contusion group were significantly lower than those in the control group. In the contusion+DFX group, SOD and GPx levels were significantly higher than those in the contusion group. In light microscopic evaluation, the contusion and contusion+DFX groups showed edema, hemorrhage, alveolar destruction, and leukocyte infiltration. However, histological scoring of the contusion+DFX group was significantly more positive than that of the contusion group. The iNOS staining in the contusion group was significantly more intensive than that in all other groups. DFX reduced iNOS staining significantly in comparison to the contusion group. This study showed that DFX reduced oxidative stress in lung contusions in rats and histopathologically ensured the recovery of the lung tissue.

## 1. Introduction

 Lung contusions are the most common form of injury caused by obtuse chest trauma in both adults and children due to things like traffic accidents and falling [1–4]. In fact, it has been known for some time that lung contusions may occur in children without any broken ribs, as the rib cage is more flexible [4, 5]. They may lead to lung pathologies that may particularly require intensive care, have a high mortality rate, and may progress similar to acute respiratory distress syndrome (ARDS) [6]. The physiopathology of lung contusions has yet to be properly understood. Experimental observations reveal that they have a highly complex inflammation process [7, 8]. Today, supportive treatments are the only treatments applied to lung contusions [1, 2]. However, experimentally applying mechanical ventilation ironically increases the inflammatory response of both lungs and systemically of other tissues [9]. We believe the use of medication for lung contusions may prevent major complications and will reduce the need for invasive practices like mechanical ventilation.

 Desferrioxamine (DFX) is a molecule with strong antioxidant properties, and it is still in use for preventing iron loading in patients who continuously receive blood transfusions, such as thalassemia patients [10–15]. It is assumed to be effective by inhibiting Fenton and Haber Weiss reactions in the tissue through iron chelation, thus reducing the formation of free hydroxyl radicals [10]. Various experimental studies on DFX in the lungs have found that it is effective in reducing oxidative stress and protecting tissues in certain cases such as chlorine inhalation and the formation of acute lung damage with major hepatectomy [11–13]. Our assessment of the literature revealed no previous use of this drug to treat lung contusions. Our hypothesis in this study is that DFX, a highly effective antioxidant, has therapeutic effects in experimental lung contusions formed in rats.

## 2. Methods

### 2.1. Animals and Experimental Protocol

 Male Sprague-Dawley rats, weighing 180–250 g, were housed in the Experimental Animals Research Laboratory, Trakya University, Turkey, under standard laboratory conditions (22 ± 1°C, 12 h light/dark cycle). The Ethical Committee of Trakya University approved all procedures and the experimental protocol concerning the animals. Rats were fed with standard rat chow and tap water ad libitum.

 Falling weight methods defined by Raghavedran et al. [16] were applied to perform pulmonary contusion. Subjects were anesthetized with 50 mg/kg intramuscular ketamine hydrochloride and 15 mg/kg intramuscular xylazine. Each rat's right hemithorax was drawn, and a 500 g metal cylinder was dropped from a 50 cm height in the supine position. In this way, trauma was standardized by applying 2.45 j of energy on the chest region according to the formula E = mgh (E: energy (joule), m: mass of cylinder (kg), g: gravity constant (9.8 m/s^2^), and h: height (meter)).

 The rats were divided into four groups (*n* = 8): (a) control, (b) control+DFX, (c) contusion, and (d) contusion+DFX. In the control+DFX and contusion+DFX groups, 100 mg/kg of DFX was given intramuscularly once a day immediately following the contusion and the day after the contusion. After 48 h in the control, control+DFX, and contusion+DFX groups, thoracotomy was done following reanesthetization. The right lung tissue of all rats was immediately biopsied. Half of the right lung was put into Bouin's solution for histopathological examination. The other half was preserved at −80°C until the biochemical parameters were analyzed.

### 2.2. Biochemical Assay

 The lung tissues were homogenized in tenfold volume of physiological saline solution by using a homogenizer (Ultra-Turrax T25, IKA; Werke 24,000 r.p.m.; Germany). The homogenate was centrifuged at 10,000 ×g for 1 h to remove debris. Clear upper supernatant was taken, and tissue analyses were carried out in this fraction. All procedures were performed at +4°C throughout the experiments. 

 Malondialdehyde (MDA) (an important indicator of oxidative stress) levels were measured according to the method used by Jain et al. [17]. The principle of the method is based on the spectrophotometric measurement of the color that appeared during thiobarbituric acid's reaction with MDA. Concentrations of thiobarbituric acid-reactive substances were calculated by the absorbance coefficient of malondialdehyde-thiobarbituric acid complex and expressed in nmol/mL. The glutathione (GSH) concentration also was measured using a spectrophotometric method [18]. After lysing samples and removing the precipitate, disodium hydrogen phosphate and DTNB solution were added, and the color formed was read at 412 nm. 

 Superoxide dismutase (SOD) and glutathione peroxidase (GPx) activities were studied on hemolysates using commercial kits (Randox Laboratories, UK) [19, 20]. Catalase (CAT) activity was measured according to Aebi's method [21]. The principle of the assay is based on the determination of the rate constant [*k*(*s* − 1)] of hydrogen peroxide decomposition by catalase enzyme. The decomposition of the substrate hydrogen peroxide was monitored spectrophotometrically at 240 nm. The rate constant was calculated from the following formula: *k* = (2.3/Δ*t*)(*a*/*b*)log⁡(*A*1/*A*2).

### 2.3. Histopathological Examination

 Lung tissue samples were fixed in 10% neutral buffered formalin immediately after removal, dehydrated in graded concentrations of ethanol, cleared in toluol, and embedded in paraffin. Sections of 6 *μ*m thickness were obtained and then stained with hematoxylin-eosin. Slides were examined by an Olympus CX31 light microscope and photographed. All histopathological changes were documented in each lung tissue, including intra-alveolar hemorrhage, alveolar edema, disruption and congestion, and leukocyte infiltration. Alveolar edema and congestion were scored on a scale from 0 to 3, where 0 = absence of pathology (<5% of maximum pathology), 1 = mild (<10%), 2 = moderate (15–20%), and 3 = severe (20–25%). Leukocyte infiltration was evaluated to determinate the severity of inflammation resulting from contusions. Each section was divided into 12 subsections, and leukocytic infiltration was examined in each of the subsections at a magnification of 400x with the following scale: 0: no extravascular leukocytes; 1: <10 leukocytes; 2: 10–45 leukocytes; 3: >45 leukocytes. The average of the numbers was used for comparison [22, 23].

### 2.4. Inducible Nitric Oxide Synthases (iNOS) Immunohistochemistry

Lung tissues were fixed in Bouin's solution, and following routine laboratory methods, embedded in paraffin. Five-micrometer paraffin tissue sections were mounted on poly-L-lysine slides. The slides were air-dried and the tissue deparaffinized. Mounted specimens were washed in 0.01 mol/L of phosphate-buffered saline (PBS). After three washes with PBS, an antigen retrieval solution (0.01 M citrate buffer, pH 6.0) was given for 15 min. at 100°C in a microwave oven, and endogenous peroxidase was eliminated by incubation in 3% H_2_O_2_ in pH 7.4 in PBS (0.01 M) for 10 min. After they were washed, the specimens were treated with a blocking serum (Labvision, TR-060-UB) at room temperature for 10 min. The sections were incubated with rabbit polyclonal anti-iNOS (Abcam, dilution 1 : 100) and reacted with tissue specimens at room temperature for 60 min. The sections were washed three times with PBS and incubated with biotinylated secondary antibody (Ultra Vision Detection System-HRP kit, Thermo, Fremont, USA). Then, streptavidin peroxidase (Ultra Vision Detection System-HRP kit, Lab Vision, Fremont, USA) was given at room temperature for 20 min. Diaminobenzidine (DAB) was used as a chromogen, and the sections were counterstained with hematoxylin. The specificity of the immunohistochemical staining was tested using PBS in the same dilutions. Control tissue sections were used as positive controls [24]. The positive cell rateunder a high powerfield (×400) was determined by the mean percentage of positive cells in six different nonoverlapping fields from each slide. iNOS-positive cell rate = (the number of iNOS-positive cells/number of total cells) × 100%.

### 2.5. Statistical Analysis

Statistical analysis of the control and the three experimental groups was compared using a one-way analysis of variance (ANOVA) and Tukey's post test. A value of *P* < 0.05 was considered statistically significant. All values were expressed as mean standard deviation. Statistical tests were performed using SPSS version 12.0 PL for Windows. 

## 3. Results

No animals were killed during this study. Animals in contusion groups were observed to have respiratory difficulties.

### 3.1. MDA and GSH Levels in Pulmonary Tissue (Table 1)

 The contusions led to a meaningful rise in the MDA level in lung tissue. MDA levels in the contusion+DFX group experienced a meaningful decline. Glutathione levels were significantly lower in the contusion group than the control group and significantly higher in the contusion+DFX group.

### 3.2. Enzyme Levels in Pulmonary Tissue (Table 2)

 GPx and SOD levels in the contusion group were significantly lower than the control group. In the contusion+DFX group, SOD and GPx levels were significantly higher than the contusion group. No meaningful between-group difference was found in catalase levels.

### 3.3. Pulmonary Histology (Table 3, Figure 1)

 While normal lung tissue was observed in control and control+DFX groups, the contusion and contusion+DFX groups showed edema, hemorrhage, alveolar destruction, and leukocyte infiltration. The histological scoring demonstrated that the scoring of the contusion+DFX group was significantly positive compared to the contusion group.

### 3.4. Pulmonary Immunohistochemical iNOS (Table 4, Figure 2)

The staining in the contusion group was significantly more intensive than all other groups. DFX reduced iNOS staining significantly when compared to the contusion group.

## 4. Discussion

 Much remains unknown about lung contusions [7, 8]. This pathology begins with a blunt chest trauma. In this study, we want to contribute to the understanding of this obscure pathology and investigate the effect of DFX. We found that alveolar rupture, edema, bronchoalveolar bleeding, subpleural bleeding, and atelectasia in the lungs in the case of lung contusions experimentally formed via obtuse chest trauma [8, 25]. Our study also identified similar histopathological findings in the contusion group. This condition also leads to deformed perfusion/ventilation rates and hypoxia. In addition, contusions trigger several inflammatory processes, paving the way for pathologies with high morbidity and mortality, such as ARDS and pneumonia [6, 7]. Inflammatory processes occurring in lung contusions are highly complex and not well understood [7]. As our study reveals, the trauma is accompanied by numerous leucocytes and macrophages in the tissue and intra-alveolar fluid. The activation of these substances leads to the production of cytokine, proteolytic enzymes, resulting in edema and capillary leaks in the alveoli [7]. Various and numerous free oxygen radicals and proteolytic and lipolytic enzymes cause cellular breakdown [7, 8, 26]. The lung contusion models in rats demonstrated that MDA levels increase in lung tissue as a final product of cellular membrane lipids' peroxidation [23, 27]. In our study, we found that the MDA levels were significantly higher in the contusion group than other groups. Similarly, previous studies conducted with experimental lung contusion models have shown the existence of oxidative stress in the pathogenesis of contusions [23, 27].

 One of the most significant pathways creating free radicals in the tissue is initiated as an oxygen molecule that receives one electron and is reduced to a superoxide radical due to leaks in the electron transport chain [28, 29]. A similar reaction is the reduction of oxygen to superoxide as the Fe^+2^ and Cu^+1^ ions lose one electron [30]. The superoxide in the tissues gains electrons slowly in the natural process, but rapidly in the presence of SOD enzyme, and, combined with two protons, transforms into hydrogen peroxide [31, 32]. In turn, hydrogen peroxide is transformed into water with the help of catalase and glutathione and is catalyzed by Gpx [33]. Hydrogen peroxide is not a strong free radical; however, combined with a superoxide radical (Haber-Weiss reaction) or in the presence of Fe^+2^ (Fenton reaction), it causes the hydroxyl radical to form much more rapidly [34]. Hydroxyl is known as a strong free radical. It causes major damage to the tissue in which it is formed [35, 36]. Our study found that contusions significantly reduce tissue GPx and SOD activities. We associate this with the expending of both enzymes in relation to the increased amounts of superoxide and hydrogen peroxide. Furthermore, we found that the GSH in the contusion group was significantly low. GSH is a tripeptide found in high concentrations both in cytosol and in alveolar in the lung [37]. It is also one of the most significant antioxidant molecules [38]. However, we were unable to identify a meaningful decline in catalase. In our experiment, killing the animals on the 48th hour may have caused the catalase enzymes to return to previous levels, as it has been found that tissue restoration begins after 48 hours in experimental lung contusions [7]. It has been shown in vitro that hydroxyl radicals develop in lesser amounts in an iron-catalyzed Fenton reaction, as DFX and other iron chelators inhibit hydroxyl radicals by removing iron from the medium [10, 39, 40]. Several studies have also shown the antioxidant effect of DFX in animal studies [11–14]. In our study, the MDA levels significantly declined in contusion groups that were administered DFX. SOD and GPx activities were found to be significantly higher compared to the contusion group. Based on these results, we can state that DFX biochemically reduces oxidative stress in lung contusions. In support of this, we found that histopathologically, DFX leads to a significant recovery of the symptoms in the lungs.

 NO is a molecule that has particular biological functions in the lungs. iNOS activity is increased, leading to the formation of high amounts of NO during various inflammatory processes in the lungs [41]. NO's reaction with free oxygen radicals creates peroxynitrite, a very strong free radical, and causes tissue damage [41, 42]. In experimental models involving inflammatory and oxidative stress in the lungs, the iNOS inhibitors have generally demonstrated positive effects [43]. Similarly, our study found a meaningful immunohistochemical increase of iNOS in the contusion group compared to the control group. In our investigation of the literature, we found no publications relating to iNOS' role in lung contusions. On the other hand, Gokce et al. [27] found an increase in NO levels in the blood of rats following contusions. In addition, it was reported that peroxynitrite increases in the lungs following contusions, which triggers inflammation and increases tissue hypoxia by causing vasoconstriction [44]. In our study, we found that DFX treatment leads to a meaningful reduction of iNOS in the tissue. We can easily interpret this case as DFX inhibits iNOS. However, it is difficult to explain this phenomenon in the existing literature. DFX also inhibits the prolyl-4-hydroxylase domain-containing enzyme (PHD) [45]. This enzyme causes the degradation of the transcription factor-1 (HIF-1) in the normoxic medium induced by hypoxia. However, PDH does not function in the hypoxic medium, and the HIF-1 accumulating in the cell nucleus realizes numerous gene transcriptions, creating a protective effect [46, 47]. In a study involving mast cells, it was found that DFX HIF-1 and NF-*κ*B increase gene expression. This effect was inhibited in the presence of iron [48]. It is understood that the iron ions are effective not only in Fenton and Haber Weiss reactions but in several other mediums. It is possible to state that even DFX's effect via iron chelating demonstrates a highly complex structure. Furthermore, under in vitro conditions, DFX and certain iron chelators have been shown to be unable to inhibit the Fenton reaction [49].

 Several studies on cell culture have found an increase in NO production by increasing HIF-1 iNOS gene expression [50]. On the contrary, it was shown that NO production in cell culture medium does not increase and does not lead to iNOS gene expression [51]. It is suggested that a significant reduction of the PaO_2_/FiO_2_ rate degrades tissue oxygenation in experimental lung contusions in rats [16, 44, 52]. Similarly, HIF activation can be expected to increase in lung contusions, in conjunction with hypoxia. Raghavendran et al. [7] showed that the pulmonary functions in lung contusions begin to normalize and the inflammation significantly improves after 48 hours. Our study reveals that oxidative stress is considerably reduced by DFX. Consequently, the mechanisms triggering reactive oxygen products and iNOS expression such as HIF subside may have caused the reduction of immunohistochemical iNOS staining. Furthermore, it was demonstrated that DFX has the function of protecting against oxidative stress under normoxic conditions through PDH inhibition, independent of HIF-1 [45].

 There are certain restrictive elements in this study. The study only includes evaluations performed within 48 hours. It may be useful to assess the findings in earlier phases to better understand DFX's effects on the different steps of contusion development.

 This study showed that DFX reduced oxidative stress in lung contusions formed in rats and histopathologically ensured the recovery of the lung tissue. We believe that active molecules are needed to prevent pathologies with high mortality rates, such as ARDS, in contusion cases with worsening clinical progress, particularly in the early period. DFX may be an effective agent in treating lung contusions, as it is a molecule with well-known usage, including the junior age group. However, more comprehensive studies are needed in this regard. It would also be beneficial to evaluate DFX's correlation with HIF-1, as DFX is a nonselective PHD inhibitor. 

## Figures and Tables

**Figure 1:**
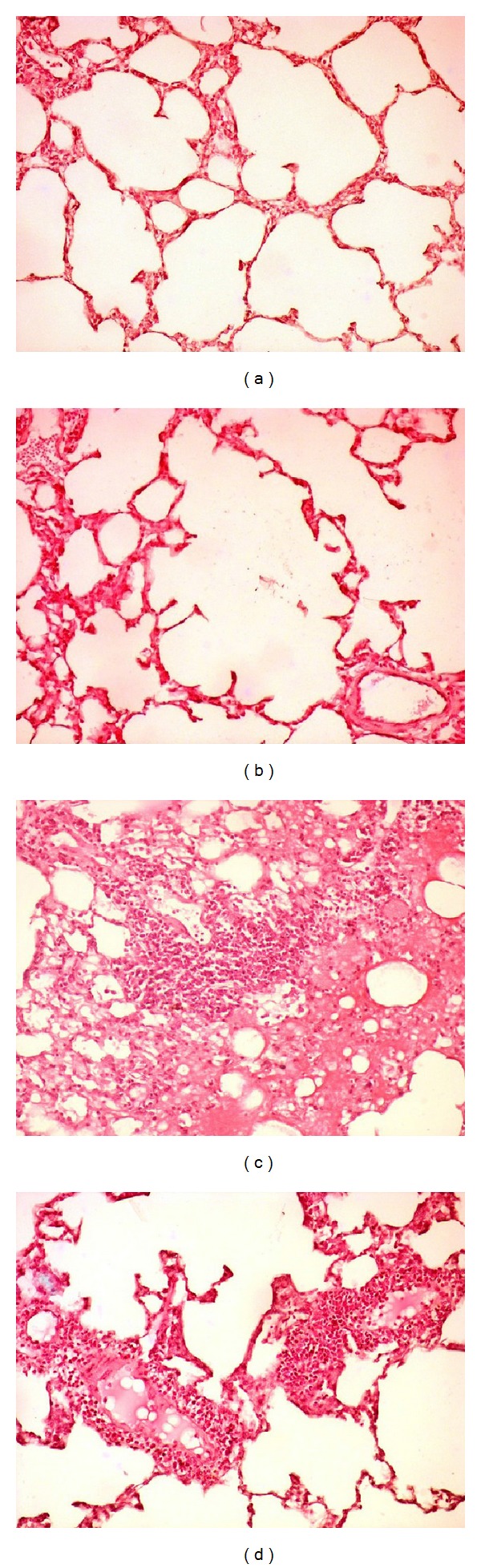
(a) In the control group, normal lung tissue architecture was seen. (b) In the control+DFX group, normal lung tissue architecture was seen. (c) In the contusion group, alveolar hemorrhage, edema, and infiltration of inflammatory cells were seen in lung tissue. (d) In the contusion+DFX group, the lung tissue of DFX treated showed a significant decrease in hemorrhage, edema, and leukocytes cells (H&E 400x).

**Figure 2:**
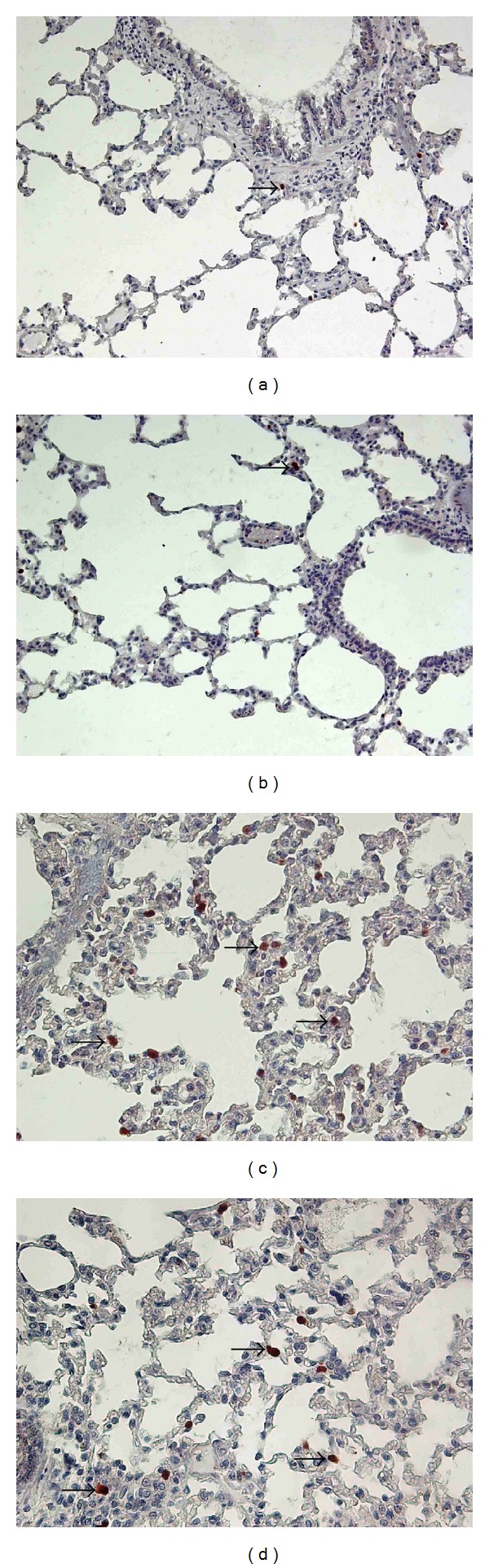
Sections of lung tissue in the different groups. (a) In the control group, normal lung tissue (arrow: iNOS positive cell). (b) In the control+DFX group, normal lung tissue (arrow: iNOS positive cell). (c) In the contusion group, there was a significant increase in the number of iNOS-positive cells (arrows) in the lung tissue. (d) In the contusion+DFX group, DFX treatment markedly reduced the number of iNOS-positive cells (arrow); (iNOS immunohistochemical staining, magnifications: (a) and (b): 400x; (c) and (d): 200x).

**Table 1:** Tissue MDA and GSH levels.

	Control	Control+DFX	Contusion	Contusion+DFX
MDA (nmol/mg tissue)	19.85 ± 2.4^b^	20.55 ± 2.5^b^	27.72 ± 4.3	23.33 ± 3.1^a^
GSH (nmol/g tissue)	290.26 ± 11.2^a^	291.30 ± 12.8^a^	269.70 ± 13.5	269.15 ± 14.7

^a^ *P* < 0.05 with respect to contusion.

^b^ *P* < 0.01 with respect to contusion.

**Table 2:** Antioxidant enzyme levels.

	Control	Control+DFX	Contusion	Contusion+DFX
CAT (kU/kg)	2312.53 ± 360.3	2425.04 ± 369.4	2550.11 ± 558.1	2462.48 ± 320.4
GPx (U/kg)	6512.54 ± 464.3^a^	6575.03 ± 677.7^a^	5437.55 ± 863.4	6325.05 ± 536.5^a^
SOD (U/mg)	223.75 ± 16.8^b^	227.50 ± 18.3^b^	187.52 ± 19.8	213.62 ± 18.5^a^

^a^ *P* < 0.05 with respect to contusion.

^b^ *P* < 0.01 with respect to contusion.

**Table 3:** Comparison of alveolar oedema/congestion scores and leukocyte infiltration of the lung tissue in each group.

Groups	Oedema/hemorrhage/alveolar disruption scores	Leukocyte infiltration
Control	0.00 ± 0.00	0.21 ± 0.13
Control+DFX	0.00 ± 0.00	0.08 ± 0.11
Contusion	2.87 ± 0.97^a,b^	3.00 ± 0.00^a,b^
Contusion+DFX	1.84 ± 0.65^a^	2.11 ± 0.63^b^

Data are presented as mean ± S.D.

^a^ *P* < 0.001 compared with Control and Control+DFX groups.

^b^ *P* < 0.001 compared with Contusion+DFX group.

**Table 4:** iNOS positive cell distribution in lung tissues.

	Control	Control+DFX	Contusion	Contusion+DFX
iNOS	0.37 ± 0.04	0.41 ± 0.06	5.05 ± 1.02^a^	3.5 ± 0.97^b^

^a^ *P* < 0.05 compared with control, control+DFX, and contusion+DFX groups.

^b^ *P* < 0.05 compared with control and control+DFX groups.
